# Association Between Long-Term Blood Pressure Patterns and Onset of Stroke: A Nested Case–Control Study of a Randomized Clinical Trial

**DOI:** 10.3390/jcdd13100493

**Published:** 2026-10-01

**Authors:** Masataka Miyama, Fumihiro Sakakibara, Mio Sakuma, Hisao Ogawa, Hirofumi Soejima, Sadanori Okada, Izuru Masuda, Masako Waki, Hideaki Jinnouchi, Yoshihiko Saito, Takeshi Morimoto

**Affiliations:** 1 Department of Data Science, Hyogo Medical University, Nishinomiya 663-8501, Japan; 2 President Office, Kumamoto University, Kumamoto 860-8556, Japan; 3 Department of Cardiovascular Medicine, Graduate School of Medical Sciences, Kumamoto University, Kumamoto 860-8556, Japan; 4 Kumamoto University Health Care Center, Kumamoto University, Kumamoto 860-8555, Japan; 5 Center for Postgraduate Training, Nara Medical University, Kashihara 634-8522, Japan; 6 Department of Endocrinology, Metabolism and Hypertension Research Institute, National Hospital Organization Kyoto Medical Center, Kyoto 612-8555, Japan; 7 Food Safety Commission of Japan, Tokyo 105-0001, Japan; 8 Department of Internal Medicine, Jinnouchi Hospital Diabetes Care Center, Kumamoto 862-0976, Japan; 9 Department of Cardiovascular Medicine, Nara Prefecture Seiwa Medical Center, Ikoma 636-0802, Japan

**Keywords:** blood pressure, cerebral infarction, intracerebral hemorrhage, stroke, nested case–control

## Abstract

Elevated blood pressure (BP) and its variability are established stroke risk factors; however, whether long-term BP patterns are associated with stroke onset in patients with type 2 diabetes remains unclear. As a post hoc analysis, we conducted a nested case–control study among 2535 patients in the Japanese Primary Prevention of Atherosclerosis with Aspirin for Diabetes (JPAD) trial. During follow-up, 117 patients developed cerebral infarction (CI), and 35 developed intracerebral hemorrhage (ICH). Controls for these cases were matched for sex, aspirin allocation, age, and diabetes duration. Systolic, diastolic, and mean BP values were collected at baseline, annually during the trial, and every two years after the completion of the trial. Sequential BP levels and coefficient of variations (CVs) were compared between cases and controls. In both CI and ICH subsets, the median intervals from the most recent BP measurement to stroke onset were similar between cases and controls (CI: 300 vs. 288 days, *p* = 0.46; ICH: 481 vs. 448 days, *p* = 0.76). The most recent BP measurements before stroke were similar between cases and controls for both CI (systolic BP, 135 vs. 133 mmHg, *p* = 0.15; diastolic BP, 74 vs. 74 mmHg, *p* = 0.85) and ICH (systolic BP, 137 vs. 134 mmHg, *p* = 0.40; diastolic BP, 75 vs. 72 mmHg, *p* = 0.17). CVs were also comparable. Among patients with type 2 diabetes enrolled in a clinical trial, we found no clear evidence of an association between long-term BP patterns and subsequent stroke onset, suggesting that other factors triggering stroke onset warrant investigation.

## 1. Introduction

Hypertension has been widely recognized as an independent modifiable risk factor for both ischemic and hemorrhagic strokes. The prevalence of hypertension among adults was approximately 40–60% in Japan as well as in the US and European countries [1,2]. Elevated systolic blood pressure (BP) contributes to endothelial dysfunction and arterial stiffness, which increases the risk of ischemic and hemorrhagic strokes [3]. A recent study showed that a 10 mmHg higher cumulative mean systolic BP was associated with increased risks of both ischemic and hemorrhagic strokes [4]. Another study showed that elevated BP was associated with a higher risk of ischemic and hemorrhagic strokes in young and middle-aged adults [5]. Hypertension affects more than 40% of patients with diabetes, and the coexistence of hypertension and type 2 diabetes synergistically increases the risk of ischemic and hemorrhagic strokes [6,7]. According to the American Heart Association guidelines, antihypertensive therapy is recommended when the average systolic BP is ≥130 mmHg or diastolic BP is ≥80 mmHg for patients with hypertension and diabetes [8].

In addition to temporal BP measurements, visit-to-visit BP variability has recently been recognized as a potential contributor to ischemic and hemorrhagic strokes. A population-based study showed that the long-term visit-to-visit BP variability was associated with an increased risk of ischemic and hemorrhagic strokes, regardless of the amplitude of variability or absolute values of BP measurements [9]. However, whether long-term BP pattern is associated with ischemic or hemorrhagic strokes onset remains unclear. Therefore, we hypothesized that long-term BP pattern could trigger the occurrence of ischemic and hemorrhagic strokes. We thus conducted a nested case–control study to evaluate the association between long-term BP patterns and the risk of ischemic and hemorrhagic strokes based on the long-term follow-up data from a large cohort of patients with type 2 diabetes without established atherosclerotic disease.

## 2. Materials and Methods

### 2.1. Study Design and Patients

We conducted a nested case–control study within the Japanese Primary Prevention of Atherosclerosis with Aspirin for Diabetes (JPAD) trial, which was a multicenter, randomized, standard-care-controlled, open-label, blinded end-point trial conducted at 163 institutions across Japan to evaluate the effect of low-dose aspirin on the prevention of cardiovascular events in patients with type 2 diabetes [10,11]. The inclusion criteria of the JPAD trial were patients with type 2 diabetes, aged between 30 and 85 years, and with the ability to provide informed consent. The exclusion criteria included those with electrocardiographic abnormalities (ischemic ST-segment depression, ST-segment elevation, or pathological Q waves); a history of coronary artery disease confirmed by coronary angiography; a history of cerebrovascular disease [cerebral infarction (CI), intracerebral hemorrhage (ICH), subarachnoid hemorrhage, or transient ischemic attack]; a history of peripheral atherosclerotic disease requiring medical treatment; atrial fibrillation; pregnancy; use of antiplatelet or antithrombotic therapy; a history of severe gastric or duodenal ulcer; severe hepatic or renal dysfunction; and allergy to aspirin. The JPAD trial enrolled patients from December 2002 through May 2005 and was completed in April 2008. After the completion, investigators continued long-term follow-up of all participants until February 2022.

The JPAD trial and the extended follow-up study were approved by the institutional review boards of participating hospitals, and written informed consent was obtained for the JPAD trial, with verbal consent for the extended follow-up study. Both the JPAD trial and the subsequent follow-up study were conducted in accordance with the latest versions of the Declaration of Helsinki and the Ethical Guidelines for Medical and Biological Research Involving Human Subjects. The current study was post hoc and thus approved by the Ethics Committee of Hyogo Medical University (approval no. 4929). During the follow-up period, the decision to prescribe low-dose aspirin was made at the discretion of each treating physician and patient.

### 2.2. Selection of Cases and Controls

Among 2535 patients enrolled in the JPAD trial, 117 (4.6%) experienced cerebral infarction (CI cases), and 35 (1.4%) experienced intracerebral hemorrhage (ICH cases) (Figure 1). All potential stroke events were adjudicated by an independent committee based on the available clinical data. Each case was matched to two controls who were under active follow-up and free from the respective event on the case’s onset date, including patients who subsequently developed the event during follow-up (Figure 2). For each matched set, the index dates for the matched controls were defined as the stroke onset date of the case. Controls were matched for sex, aspirin allocation, and age (±3 years). Among eligible controls, two with the closest diabetes duration to the corresponding case were selected. If the diabetes duration of a case was unavailable, duration-based matching was omitted. Consequently, 234 CI controls and 70 ICH controls were selected (Figure 1). As a result, 117 CI cases and 234 CI controls consisted of the CI subset, and 35 ICH cases and 70 ICH controls consisted of the ICH subset.

### 2.3. Measurements

Baseline demographic and clinical characteristics were obtained upon enrolment in the JPAD trial, including age, sex, diabetes duration, medical history, and medications. Laboratory data included HbA1c, lipid profiles, serum creatinine, and a complete blood count. BP was measured in the seated position after 1–2 min of rest using an automatic sphygmomanometer, with the forearm supported on a table and the cuff positioned at heart level. BP data (systolic, diastolic, and mean) were collected at baseline, annually during the JPAD trial, and every 2 years during the follow-up period (Figure 2). BP data were sequentially numbered backward from the onset of stroke to the past (past 1, past 2, past 3, etc.). No BP measurements obtained after the onset of stroke were included in the analysis.

### 2.4. Statistical Analysis

Patient characteristics were compared between cases and controls separately in the CI and ICH subsets. Categorical variables are presented as numbers (percentages) and continuous variables as means ± SD or medians (interquartile range). Depending on the data distribution, continuous variables were compared using Student’s *t*-test or the Wilcoxon rank-sum test, as appropriate. Patients with missing values for any of the selected variables were excluded from the corresponding analyses.

BP data immediately preceding the onset of stroke (past 1) were compared between cases and controls in each subset using the Student’s *t*-test. In addition, BP data at each follow-up (past 2, past 3, etc.) were analyzed similarly. Matched pairs with missing BP data for either the case or control were excluded from the analysis. To account for the matched design, we first calculated the difference [the measurements preceding the onset of stroke of the case minus the measurements of its two matched controls] for each matched set. We then estimated the mean of these differences together with the corresponding 95% confidence interval. Among patients with at least two BP assessments during follow-up, the coefficient of variation (CV), defined as the standard deviation divided by the mean during the follow-up period for each patient, was calculated and compared between cases and controls using Student’s *t*-test.

Because some patients had a small number of BP measurements, and the long-term BP patterns were uncertain, we performed sensitivity analyses excluding patients with two BP measurements before stroke onset.

All statistical analyses were performed using JMP 18.2 (SAS Institute Inc., Cary, NC, USA). All reported *p* values were two-tailed, and *p* < 0.05 was considered statistically significant. No adjustments for multiple comparisons were performed because of the exploratory nature of this study.

## 3. Results

### 3.1. Baseline Characteristics

In the CI subset, the mean age was 67 ± 9.4 years, and 64% were male (Table 1). The baseline systolic BP was significantly higher in cases than controls (138 mmHg vs. 135 mmHg, *p* = 0.0497), whereas the baseline diastolic BP was comparable (78 mmHg vs. 77 mmHg, *p* = 0.32). The median diabetes duration was similar between cases and controls (8.3 years vs. 7.7 years, *p* = 0.82). Diabetic nephropathy was more prevalent in cases (18% vs. 8%, *p* = 0.01), and the use of multiple antihypertensive agents was also more common in cases (33% vs. 22%, *p* = 0.03).

In the ICH subset, the mean age was 66 ± 11 years, and 49% were male (Table 2). The baseline systolic and diastolic BP were comparable between cases and controls (137 mmHg vs. 139 mmHg, *p* = 0.82; 77 mmHg vs. 77 mmHg, *p* = 0.88). The median diabetes duration did not differ significantly (8.0 years vs. 7.0 years, *p* = 0.95).

When comparing cases between the CI and ICH cases, the CI cases tended to be predominantly male (64% vs. 49%, *p* = 0.10) (Table 3). Although the systolic and diastolic BP levels at baseline were numerically similar between the cases, the CI cases had a higher prevalence of hypertension (66% vs. 51%, *p* = 0.12) and were more likely to be receiving antihypertensive therapy (59% vs. 49%, *p* = 0.28). Diabetes control at baseline was comparable between the cases (median HbA1c: 7.3% vs. 7.3%, *p* = 0.52).

### 3.2. Longitudinal Blood Pressure Control

In the CI subset, the median interval from the most recent BP measurement to stroke onset was similar between the cases and controls (300 days vs. 288 days, *p* = 0.46) (Table 4). The most recent systolic, diastolic and mean BP values before onset were also comparable (135 mmHg vs. 133 mmHg, *p* = 0.15; 74 mmHg vs. 74 mmHg, *p* = 0.85; 94 mmHg vs. 94 mmHg, *p* = 0.39). The 95% confidence intervals for mean differences in systolic and diastolic BP values included the null (0).

Longitudinal systolic BPs were numerically higher in cases than in controls across follow-up assessments in the CI subset, while the differences were not statistically significant (Figure 3a). In both cases and controls, systolic BP was generally well controlled below 140 mmHg. Diastolic BP was controlled similarly between cases and controls throughout the follow-up period (Figure 3b). The median number of BP measurements used to calculate CV was 5 (IQR, 2–7) for both cases and controls (Table 4). The CVs for systolic and diastolic BP during the follow-up period did not differ significantly (0.07 vs. 0.07, *p* = 0.88; 0.09 vs. 0.09, *p* = 0.45). The results of the sensitivity analyses were consistent with those of the primary analyses (Supplementary Table S1).

In the ICH subset, the median interval from the most recent BP measurement to stroke onset was comparable between cases and controls (481 days vs. 448 days, *p* = 0.76) (Table 4). The most recent systolic, diastolic, and mean BP values before onset did not differ significantly (137 mmHg vs. 134 mmHg, *p* = 0.40; 75 mmHg vs. 72 mmHg, *p* = 0.17; 96 mmHg vs. 93 mmHg, *p* = 0.19). The 95% confidence intervals for mean differences in systolic and diastolic BP values included the null (0).

In the ICH subset, the systolic BP showed no significant difference between cases and controls at any follow-up point (Figure 4a). However, the diastolic BP tended to be higher in cases, with a statistically significant difference observed at past 2 (*p* = 0.03) (Figure 4b). The median number of BP measurements used to calculate CV was five (IQR, 2–8) for cases and five (IQR, 2–7) for controls (Table 4). The CVs for systolic and diastolic BP during the follow-up period were also comparable (0.08 vs. 0.08, *p* = 0.86; 0.09 vs. 0.10, *p* = 0.55). The results of the sensitivity analyses were consistent with those of the primary analyses, and the 95% confidence interval of difference in diastolic BP did not include zero (Supplementary Table S1).

## 4. Discussion

In this nested case–control study of patients with type 2 diabetes, we found no clear evidence of an association between long-term BP patterns and the subsequent occurrence of either CI or ICH. In addition to the comparable BP values of the most recent measurements between cases and controls in both CI and ICH subsets, the longitudinal measurements of both systolic and diastolic BP values were similar between patients with and without CI. This finding was also observed for the ICH subset. The CVs for systolic and diastolic BP during the follow-up period were comparable between cases and controls in both CI and ICH subsets.

We tried to investigate the impact of BP control on CI and ICH occurrence in a large cohort of Japanese patients who were under medical care in a trial rather than the general public, and BP control was not identified as a trigger for the onset of either CI or ICH. Numerous studies have reported an association between higher BP values during follow-up and the risk of CI and ICH. In the Action to Control Cardiovascular Risk in Diabetes (ACCORD), which focused on patients with diabetes, intensive BP lowering was associated with a 41% reduction in both ischemic and hemorrhagic stroke risks, although these strokes were not the primary endpoints, and the results were based on relatively few events [12]. In the Systolic Blood Pressure Intervention Trial (SPRINT), which enrolled participants without diabetes or prior stroke, the incidence of ischemic and hemorrhagic strokes did not differ between groups with lower and higher BP control during the follow-up [13]. Wang et al. reported that the general public with systolic BP controlled within 110–119 mmHg was associated with a significantly lower risk of ICH than those with systolic BP controlled within 130–139 mmHg based on the UK Biobank, a large prospective cohort study in the UK [14]. It also showed that diastolic BP controlled within 70-–79 mmHg was associated with a lower risk of ICH compared to a diastolic BP within 80–89 mmHg.

In contrast to these previous studies, which reported associations between long-term BP patterns and the occurrence of ischemic and hemorrhagic stroke, our study, which used a nested case–control design, did not show differences in BP management between cases and controls. The previous sub-analysis of the same cohort as in this study demonstrated that the baseline BP level was associated with CI [15], and the current analyses compared the preceding BP control between those with and without events after the matching of the baseline. Although the cases with CI and ICH were compatible with the previous study with the total cohort, the patients who did not match the cases were considered to have relatively lower BP levels in this analysis. Our hypothesis was that cases had poor BP control in terms of absolute value or variability, but this was not confirmed. Although the baseline BP values were not matched, the difference in systolic and diastolic BP between cases and controls was 2–3 mmHg in this analysis. Because the previous study grouped the cohort with baseline BP values, the difference in systolic BP between higher and lower BP groups was 23 mmHg [15]. The approximately 20 mmHg higher baseline BP indicated that the higher incidence of CI was driven by the large differences in BP control, which indicated that poor BP control became the risk factor for CI over the long term. On the other hand, our study suggested that BP levels or the variability of patients with CI or ICH were not higher than their counterparts on average. The poor variability or surge of BP before the occurrence of stroke was not observed. These observations suggested that BP control itself might not directly trigger the occurrence of stroke among patients with relatively well-controlled BP who were enrolled in clinical trial, generating the hypothesis that the onset might be triggered by factors other than BP.

The risk factors for ischemic and hemorrhagic strokes were of two types generally; those were non-modifiable or irreversible risk factors and modifiable or reversible risk factors [16]. Non-modifiable factors included age, sex, ethnicity and genetics [16]. On the other hand, the INTERSTROKE study demonstrated that 10 potentially modifiable risk factor including hypertension, diabetes, dyslipidemia, coexisting cardiovascular diseases, obesity, smoking, alcohol intake, unhealthy diet, physical inactivity and psychosocial stress were collectively associated with about 90% of the population attribute risk of stroke worldwide [17]. These findings highlighted that BP was one of the contributors to predisposing strokes, but the onset of stroke was driven by multiple factors [17]. Our analysis was thus consistent with this theory that the BP level or its variability was not associated with the onset of stroke. This distinction was explored by the nested case–control study of a randomized clinical trial, which focused on the efficacy of aspirin [10]. In this circumstance, patients received optimal medical management and maintain well-controlled BP levels in both cases and controls, thereby attenuating the individual effects of BP control. Other factors could also trigger an onset in patients with a predisposed status to stroke. Other temporal factors, such as mental and physical stress, short-term medication adherence, or other physical problems could trigger the onset of stroke [18]. However, these factors were not collected in this study and could not be assessed. Further research with detailed information of preceding physical status is warranted to clarify the mechanism triggering the onset of stroke in high-risk patients.

Despite several strengths, including a large number of patients with long-term follow-up and the repeated measurement of BP, this study had several limitations. First, even at the most recent follow-up, about one year had already passed. Thus, it was inevitable that the BP level just before onset could be different from the recorded data in the study. Second, estimates of BP variability based on a limited number and irregular timing of BP measurements should be interpreted with caution, and the exclusion of patients with only one BP measurement could introduce selection bias. Third, comparing cases and controls separately across multiple time points did not account for within-subject correlation or varying sample sizes. Because this study was a post hoc analysis of a randomized clinical trial and did not plan to measure the BP in the fixed time frame, the changing sample composition over time should be considered when interpreting our findings. Fourth, patients’ risk profiles at stroke onset could be different from those at enrollment in this long-term follow-up study. In addition, incomplete longitudinal data limited comprehensive adjustment for these time-varying factors. Although the nested case–control design was assumed to balance the important baseline characteristics, it was inevitable that other factors could become confounding factors and mask the actual relationship. Fifth, there were multiple comparisons of systolic and diastolic BP across numerous time points, which increased the risk of type I error. Therefore, the statistical significance for diastolic BP in the ICH subset should be interpreted cautiously. Sixth, the sample size was too small to obtain the reliable estimates, especially for the ICH subset. Therefore, the findings should be interpreted with caution, due to the limited statistical power. Finally, our analysis was conducted in a cohort of patients who participated in a randomized clinical trial. Such patients are generally more closely monitored and could have better risk factor control than those seen in daily clinical practice. Thus, the generalizability of our findings to the broader population should be examined.

## 5. Conclusions

Among patients with type 2 diabetes enrolled in a clinical trial, we found no clear evidence of an association between long-term BP patterns and subsequent stroke onset. These observations suggested that, in this relatively well-treated population, long-term office BP levels and visit-to-visit variability were not substantially different between cases and matched controls, generating the hypothesis that stroke onset in this population might be influenced by factors other than long-term BP patterns.

## Figures and Tables

**Figure 1:**
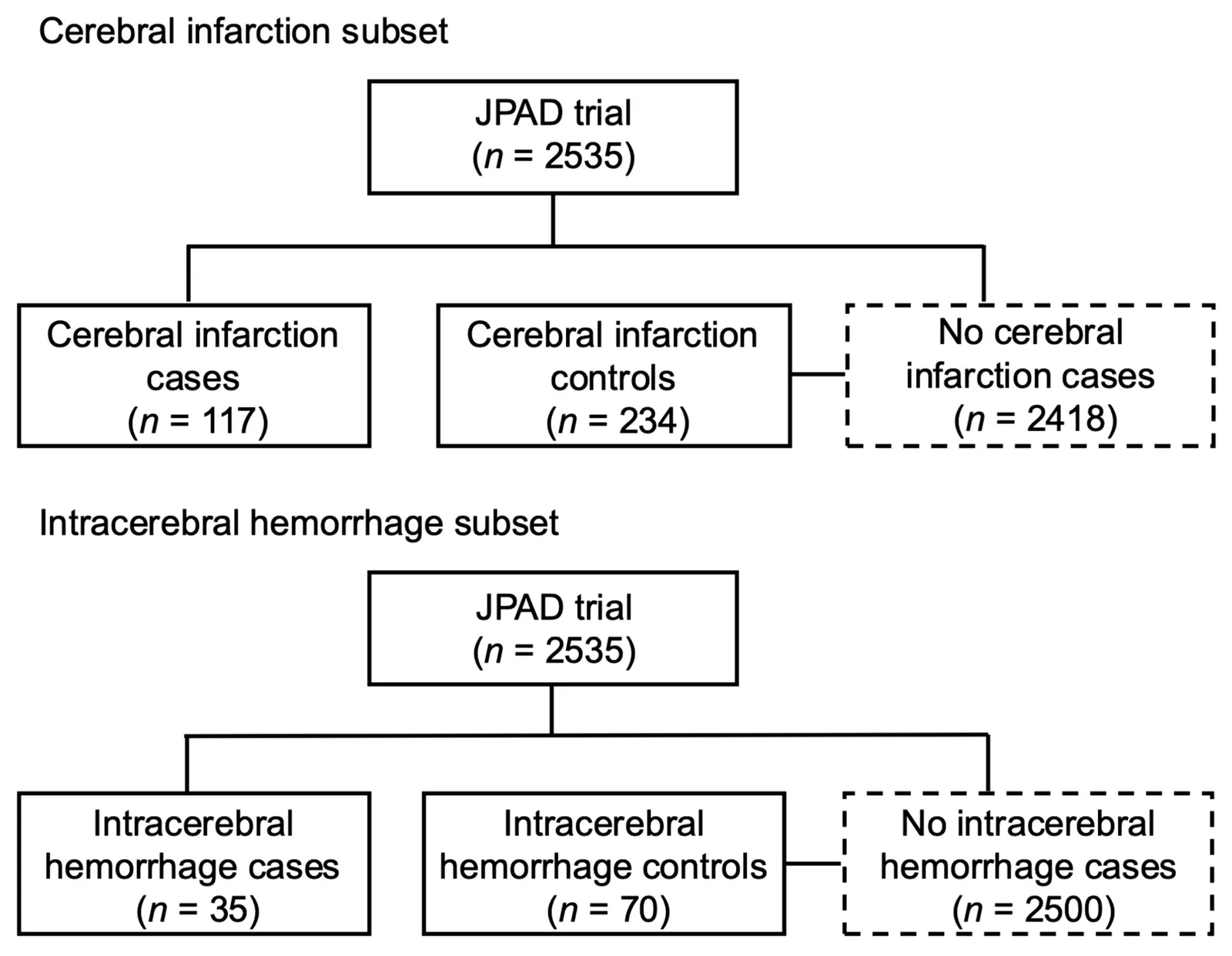
Study flow chart. JPAD, Japanese Primary Prevention of Atherosclerosis with Aspirin for Diabetes. The dashed boxes indicated patients without the respective event, from whom two controls were selected for each case.

**Figure 2:**
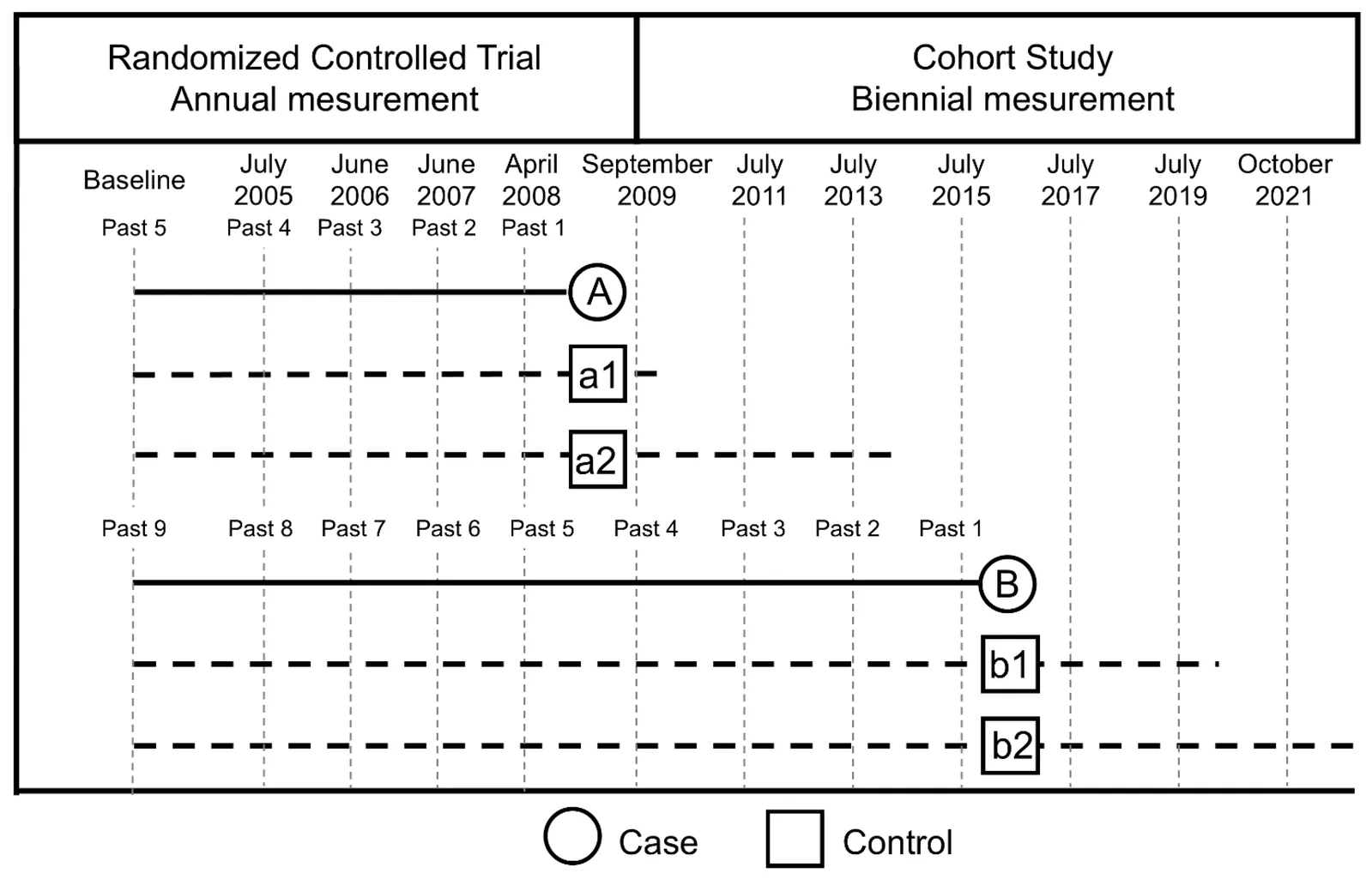
Nested case–control study, with timing of BP measurement. For Case A, controls (a1 and a2) were selected by matching for aspirin allocation, sex, age and duration of diabetes. BP data were sequentially numbered backward from the onset of stroke to the past (past 1, past 2, past 3, past 4). For Case B, controls (b1 and b2) were selected by matching for aspirin allocation, sex, age and duration of diabetes. BP data were sequentially numbered backward from the onset of stroke to the past (past 1, past 2, past 3, past 4, past 5, past 6, past 7, past 8). A maximum of 12 BP measurement time points were available for the cases with an onset date after 1 October 2021. No BP measurements obtained after the onset of stroke were included in the analysis. BP, blood pressure; CI, cerebral infarction; ICH, intracerebral hemorrhage.

**Figure 3:**
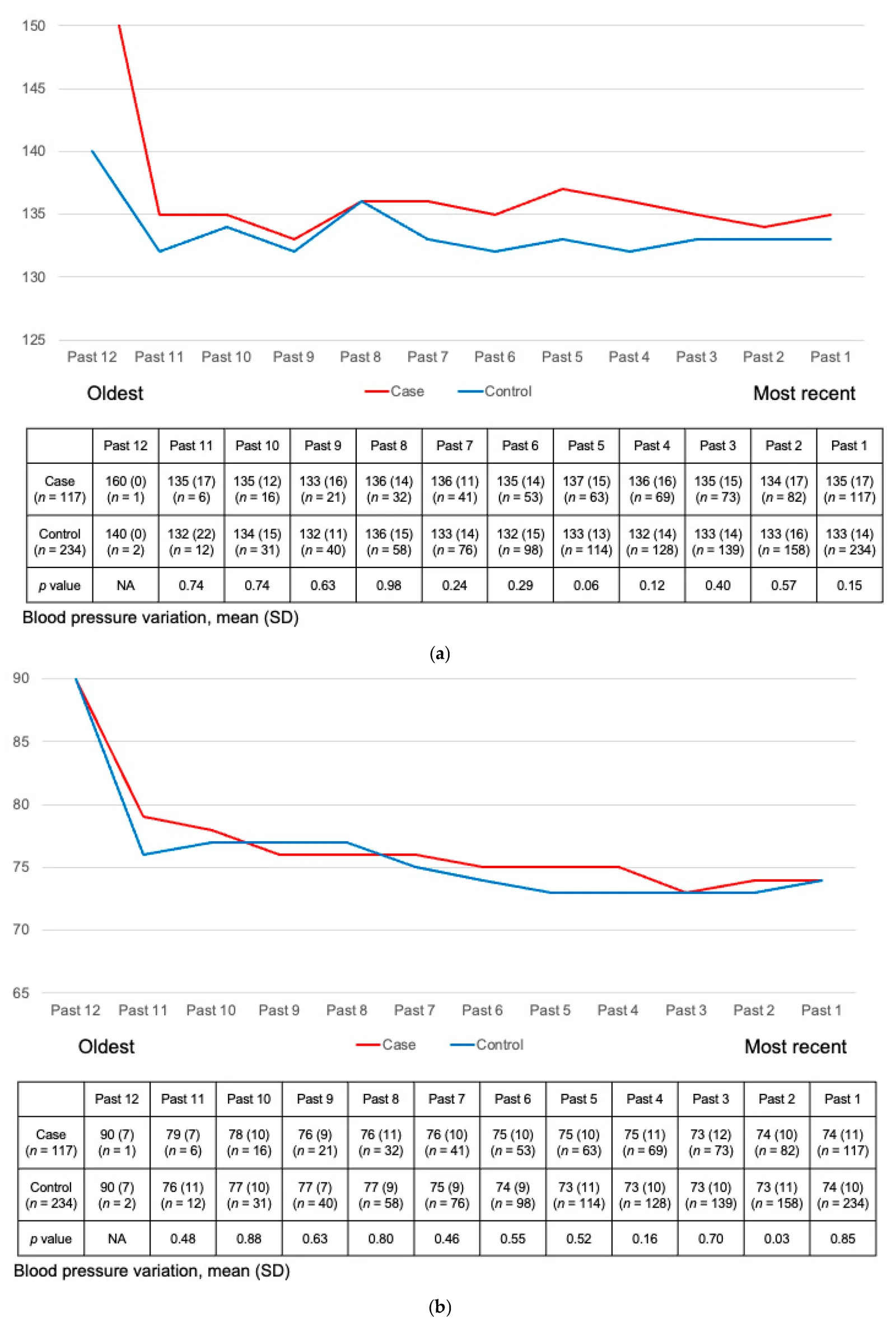
Blood pressure measurements at each follow-up point for cerebral infarction. (**a**) Systolic blood pressure; (**b**) Diastolic blood pressure. NA, not applicable; SD, standard deviation.

**Figure 4:**
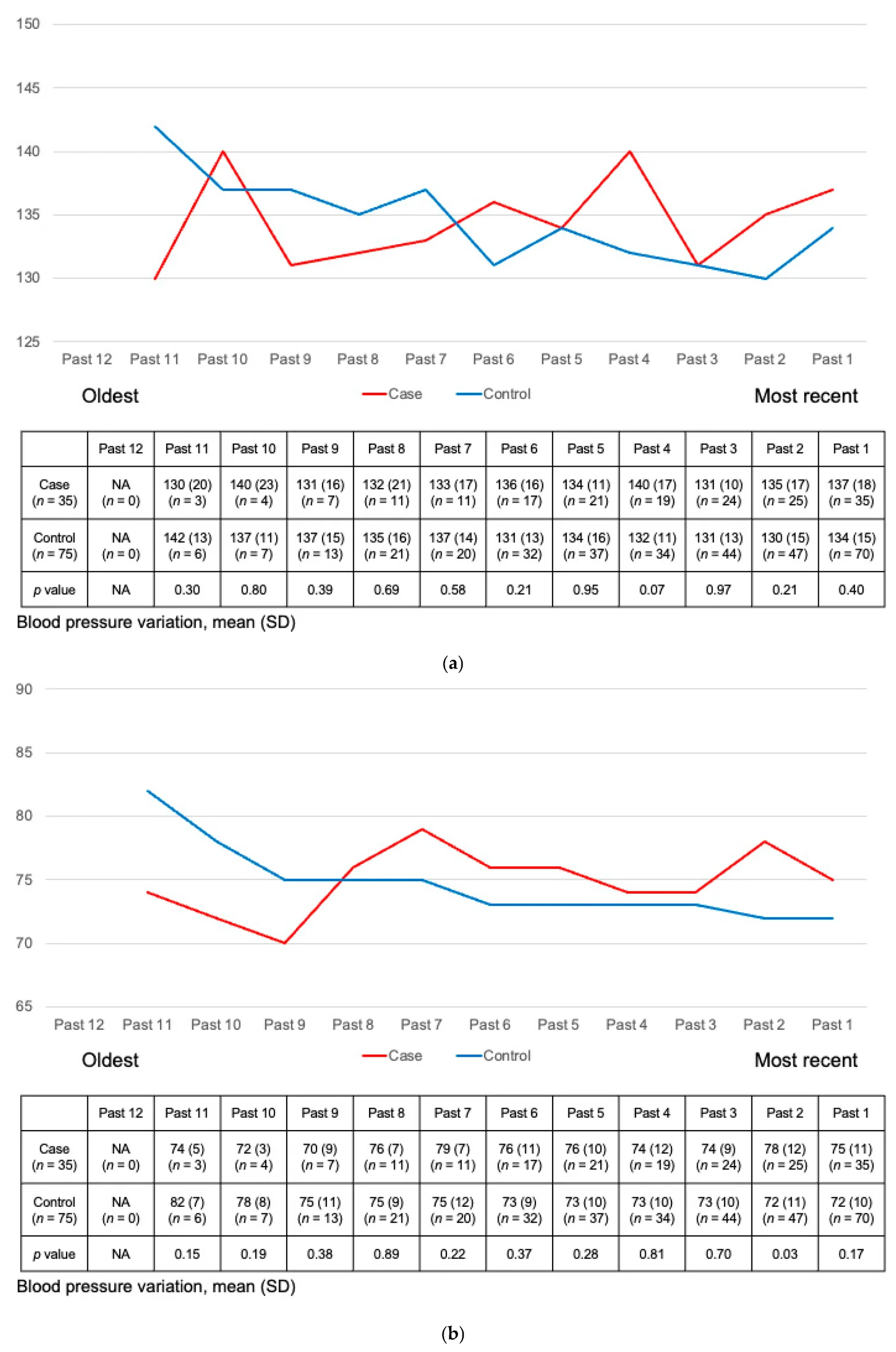
Blood pressure measurements at each follow-up point for intracerebral hemorrhage. (**a**) Systolic blood pressure; (**b**) diastolic blood pressure. NA, not applicable; SD, standard deviation.

**Table 1:** Comparison of baseline patient characteristics between CI cases and controls.

Variables	CI Case (*n* = 117)	CI Control (*n* = 234)	*p* Value
Age—years, mean ± SD	67 ± 9.4	67 ± 9.3	0.98
Men, *n* (%)	75 (64)	150 (64)	1.00
BMI—kg/m^2^, mean ± SD	25 ± 3.4	24 ± 3.3	0.24
Systolic BP—mmHg, mean ± SD	138 ± 15	135 ± 15	0.0497
Diastolic BP—mmHg, mean ± SD	78 ± 9.9	77 ± 9.5	0.32
Current smoker, *n* (%)	25 (21)	43 (18)	0.51
Past smoker, *n* (%)	27 (23)	66 (28)	0.30
Medical history			
Hypertension, *n* (%)	77 (66)	143 (61)	0.39
Hyperlipidemia, *n* (%)	61 (52)	119 (51)	0.82
Gastric ulcer, *n* (%)	9 (7.7)	26 (11)	0.31
Duration of DM—years, median [IQR]	8.3 [3.6–14.5] (*n* = 105)	7.7 [4.4–13.8] (*n* = 224)	0.82
Diabetic microvascular complication			
Diabetic retinopathy, *n* (%)	23 (20)	30 (13)	0.09
Diabetic nephropathy, *n* (%)	21 (18)	19 (8)	0.01
Diabetic neuropathy, *n* (%)	15 (13)	33 (14)	0.74
Family history			
Diabetes mellitus, *n* (%)	38 (32)	100 (43)	0.06
Ischemic heart disease, *n* (%)	12 (10)	29 (12)	0.56
Stroke, *n* (%)	29 (25)	45 (19)	0.23
Laboratory work			
HbA1c—%, median [IQR]	7.3 [6.7–8.5]	7.2 [6.5–7.9]	0.07
Fasting plasma glucose level—mg/dL, median [IQR]	141 [115–179] (*n* = 106)	140 [115–179] (*n* = 213)	0.11
Fasting triglyceride—mg/dL, median [IQR]	130 [87–179] (*n* = 110)	106 [73–154] (*n* = 225)	0.01
Total cholesterol—mg/dL, median [IQR]	201 [176–222] (*n* = 117)	197 [178–217] (*n* = 232)	0.41
LDL cholesterol—mg/dL, median [IQR]	122 [100–140] (*n* = 103)	115 [99–139] (*n* = 214)	0.44
HDL cholesterol—mg/dL, median [IQR]	50 [43–61] (*n* = 109)	54 [44–63] (*n* = 219)	0.25
BUN—mg/dL, median [IQR]	15 [13–18] (*n* = 112)	16 [13–19] (*n* = 220)	0.23
Creatinine—mg/dL, median [IQR]	0.80 [0.70–0.91] (*n* = 117)	0.80 [0.60–0.90] (*n* = 230)	0.31
eGFR—mL/min/1.73 m^2^, median [IQR]	67 [57–81] (*n* = 117)	71 [60–83] (*n* = 230)	0.26
CRP—mg/dL, median [IQR]	0.13 [0.10–0.30] (*n* = 72)	0.10 [0.05–0.20] (*n* = 148)	0.002
WBC—1000/uL, median [IQR]	6.2 [4.9–7.2] (*n* = 108)	5.8 [4.9–7.0] (*n* = 218)	0.36
RBC—1000/uL, median [IQR]	451 [415–488] (*n* = 108)	454 [426–484] (*n* = 219)	0.59
Hb—mg/dL, mean ± SD	14 ± 1.5 (*n* = 108)	14 ± 1.3 (*n* = 218)	0.83
Proteinuria ≥ (±), *n* (%)	23 (20) (*n* = 115)	30 (13) (*n* = 232)	0.08
Proteinuria ≥ (+), *n* (%)	19 (17) (*n* = 115)	24 (10) (*n* = 232)	0.10
Medication			
Aspirin allocation, *n* (%)	56 (48)	112 (48)	1.00
Antihyperglycemic drugs			
Insulin, *n* (%)	20 (17)	27 (12)	0.15
Sulfonylureas, *n* (%)	70 (60)	139 (59)	0.93
α-glucosidase inhibitors, *n* (%)	28 (24)	77 (33)	0.08
Biguanides, *n* (%)	17 (15)	34 (15)	1.00
Thiazolidines, *n* (%)	8 (6.8)	12 (5.1)	0.51
Antihypertensive drugs			
Calcium-channel blockers, *n* (%)	52 (44)	93 (40)	0.40
β-blockers, *n* (%)	10 (8.6)	9 (3.9)	0.07
α-blockers, *n* (%)	3 (2.6)	9 (3.9)	0.53
ACE inhibitors, *n* (%)	23 (20)	30 (13)	0.09
ARB, *n* (%)	26 (22)	49 (21)	0.78
Nitrate, *n* (%)	1 (0.9)	1 (0.4)	1.00
Antihypertensive drugs ≥ 1, *n* (%)	69 (59)	128 (55)	0.45
Antihypertensive drugs ≥ 2, *n* (%)	39 (33)	53 (22)	0.03
Statin, *n* (%)	23 (20)	59 (25)	0.25
Anti-ulcer drug, *n* (%)	14 (12)	33 (14)	0.58

ACE, angiotensin converting enzyme; ARB, angiotensin receptor blocker; BP, blood pressure; BMI, body mass index; BUN, blood urea nitrogen; CI, cerebral infarction; CRP, C-reactive protein; DM, diabetes mellitus; eGFR, estimated glomerular filtration rate; Hb, hemoglobin; HDL, high-density lipoprotein; IQR, interquartile range; LDL, low-density lipoprotein; RBC, red blood cell; SD, standard deviation; WBC, white blood cell.

**Table 2:** Comparison of baseline patient characteristics between ICH cases and controls.

Variables	ICH Case (*n* = 35)	ICH Control (*n* = 70)	*p* Value
Age—years, mean ± SD	66 ± 11	66 ± 11	1.00
Men, *n* (%)	17 (49)	34 (49)	1.00
BMI—kg/m^2^, mean ± SD	24 ± 3.3	25 ± 3.7	0.40
Systolic BP—mmHg, mean ± SD	137 ± 19	139 ± 17	0.82
Diastolic BP—mmHg, mean ± SD	77 ± 9.6	77 ± 9.2	0.88
Current smoker, *n* (%)	9 (26)	8 (11)	0.06
Past smoker, *n* (%)	7 (20)	15 (21)	0.87
Medical history			
Hypertension, *n* (%)	18 (51)	45 (64)	0.20
Hyperlipidemia, *n* (%)	17 (49)	36 (51)	0.78
Gastric ulcer, *n* (%)	3 (8.6)	3 (4.3)	0.37
Duration of DM—years, median [IQR]	8.0 [3.1–13.2] (*n* = 33)	7.0 [3.9–14.3] (*n* = 70)	0.95
Diabetic microvascular complication			
Diabetic retinopathy, *n* (%)	8 (23)	10 (14)	0.27
Diabetic nephropathy, *n* (%)	6 (17)	4 (6)	0.06
Diabetic neuropathy, *n* (%)	5 (14)	12 (17)	0.70
Family history			
Diabetes mellitus, *n* (%)	19 (54)	28 (40)	0.17
Ischemic heart disease, *n* (%)	4 (11)	9 (13)	0.83
Stroke, *n* (%)	7 (20)	9 (13)	0.33
Laboratory work			
HbA1c—%, median [IQR]	7.3 [6.7–8.0]	7.4 [6.7–8.2]	0.52
Fasting plasma glucose level—mg/dL, median [IQR]	131 [111–162] (*n* = 33)	132 [121–157] (*n* = 64)	0.75
Fasting triglyceride—mg/dL, median [IQR]	113 [86–160] (*n* = 32)	118 [87–142] (*n* = 65)	0.96
Total cholesterol—mg/dL, median [IQR]	202 [175–218] (*n* = 35)	197 [181–227] (*n* = 68)	0.44
LDL cholesterol—mg/dL, median [IQR]	107 [92–133] (*n* = 29)	116 [99–143] (*n* = 60)	0.21
HDL cholesterol—mg/dL, median [IQR]	56 [44–64] (*n* = 33)	58 [48–66] (*n* = 61)	0.57
BUN—mg/dL, median [IQR]	16 [13–19] (*n* = 33)	15 [12–17] (*n* = 62)	0.21
Creatinine—mg/dL, median [IQR]	0.80 [0.56–1.00] (*n* = 35)	0.70 [0.60–0.90] (*n* = 69)	0.80
eGFR—mL/min/1.73 m^2^, median [IQR]	72 [60–91] (*n* = 35)	73 [63–86] (*n* = 69)	0.91
CRP—mg/dL, median [IQR]	0.10 [0.03–0.20] (*n* = 26)	0.10 [0.03–0.20] (*n* = 44)	0.90
WBC—1000/uL, median [IQR]	6.3 [5.2–7.6] (*n* = 34)	5.9 [5.0–6.6] (*n* = 60)	0.44
RBC—1000/uL, median [IQR]	443 [418–497] (*n* = 34)	451 [423–488] (*n* = 60)	0.57
Hb—mg/dL, mean ± SD	14 ± 1.8 (*n* = 33)	14 ± 1.4 (*n* = 59)	0.70
Proteinuria ≥ (±), *n* (%)	8 (23) (*n* = 35)	7 (10) (*n* = 67)	0.09
Proteinuria ≥ (+), *n* (%)	7 (20) (*n* = 35)	7 (10) (*n* = 67)	0.37
Medication			
Aspirin allocation, *n* (%)	15 (43)	30 (43)	1.00
Antihyperglycemic drugs			
Insulin, *n* (%)	5 (14)	7 (10)	0.53
Sulfonylureas, *n* (%)	20 (57)	48 (69)	0.25
α-glucosidase inhibitors, *n* (%)	14 (40)	23 (33)	0.47
Biguanides, *n* (%)	6 (17)	12 (17)	1.00
Thiazolidines, *n* (%)	1 (2.9)	3 (4.3)	1.00
Antihypertensive drugs			
Calcium-channel blockers, *n* (%)	10 (29)	23 (33)	0.66
β-blockers, *n* (%)	2 (5.7)	3 (4.3)	1.00
α-blockers, *n* (%)	3 (8.6)	0 (0)	0.03
ACE inhibitors, *n* (%)	5 (14)	11 (16)	0.85
ARB, *n* (%)	7 (20)	20 (29)	0.34
Nitrate, *n* (%)	0 (0)	2 (2.9)	0.31
Antihypertensive drugs ≥ 1, *n* (%)	17 (49)	39 (56)	0.49
Antihypertensive drugs ≥ 2, *n* (%)	9 (26)	17 (24)	0.87
Statin, *n* (%)	12 (34)	17 (24)	0.28
Anti-ulcer drug, *n* (%)	0 (0)	5 (7)	0.17

ACE, angiotensin converting enzyme; ARB, angiotensin receptor blocker; BP, blood pressure; BMI, body mass index; BUN, blood urea nitrogen; CRP, C-reactive protein; DM, diabetes mellitus; eGFR, estimated glomerular filtration rate; Hb, hemoglobin; HDL, high-density lipoprotein; ICH, intracerebral hemorrhage; IQR, interquartile range; LDL, low-density lipoprotein; RBC, red blood cell; SD, standard deviation; WBC, white blood cell.

**Table 3:** Comparison of baseline patient characteristics between CI cases and ICH cases.

Variables	CI Case (*n* = 117)	ICH Case (*n* = 35)	*p* Value
Age—years, mean ± SD	67 ± 9.4	66 ± 11	0.49
Men, *n* (%)	75 (64)	17 (49)	0.10
BMI—kg/m^2^, mean ± SD	25 ± 3.4	24 ± 3.3	0.81
Systolic BP—mmHg, mean ± SD	138 ± 15	137 ± 19	0.66
Diastolic BP—mmHg, mean ± SD	78 ± 9.9	77 ± 9.6	0.38
Current smoker, *n* (%)	25 (21)	9 (26)	0.59
Past smoker, *n* (%)	27 (23)	7 (20)	0.70
Medical history			
Hypertension, *n* (%)	77 (66)	18 (51)	0.12
Hyperlipidemia, *n* (%)	61 (52)	17 (49)	0.71
Gastric ulcer, *n* (%)	9 (7.7)	3 (8.6)	0.87
Duration of DM—years, median [IQR]	8.3 [3.6–14.5] (*n* = 105)	8.0 [3.1–13.2] (*n* = 33)	0.80
Diabetic microvascular complication			
Diabetic retinopathy, *n* (%)	23 (20)	8 (23)	0.68
Diabetic nephropathy, *n* (%)	21 (18)	6 (17)	0.91
Diabetic neuropathy, *n* (%)	15 (13)	5 (14)	0.82
Family history			
Diabetes mellitus, *n* (%)	38 (32)	19 (54)	0.02
Ischemic heart disease, *n* (%)	12 (10)	4 (11)	0.84
Stroke, *n* (%)	29 (25)	7 (20)	0.56
Laboratory work			
HbA1c—%, median [IQR]	7.3 [6.7–8.5]	7.3 [6.7–8.0]	0.52
Fasting plasma glucose level—mg/dL, median [IQR]	141 [115–179] (*n* = 106)	131 [111–162] (*n* = 33)	0.34
Fasting triglyceride—mg/dL, median [IQR]	130 [87–179] (*n* = 110)	113 [86–160] (*n* = 32)	0.34
Total cholesterol—mg/dL, median [IQR]	201 [176–222] (*n* = 117)	202 [175–218] (*n* = 35)	0.59
LDL cholesterol—mg/dL, median [IQR]	122 [100–140] (*n* = 103)	107 [92–133] (*n* = 29)	0.15
HDL cholesterol—mg/dL, median [IQR]	50 [43–61] (*n* = 109)	56 [44–64] (*n* = 33)	0.28
BUN—mg/dL, median [IQR]	15 [13–18] (*n* = 112)	16 [13–19] (*n* = 33)	0.39
Creatinine—mg/dL, median [IQR]	0.80 [0.70–0.91] (*n* = 117)	0.80 [0.56–1.00] (*n* = 35)	0.18
eGFR—mL/min/1.73 m^2^, median [IQR]	67 [57–81] (*n* = 117)	72 [60–91] (*n* = 35)	0.27
CRP—mg/dL, median [IQR]	0.13 [0.10–0.30] (*n* = 72)	0.10 [0.03–0.20] (*n* = 26)	0.01
WBC—1000/uL, median [IQR]	6.2 [4.9–7.2] (*n* = 108)	6.3 [5.2–7.6] (*n* = 34)	0.80
RBC—1000/uL, median [IQR]	451 [415–488] (*n* = 108)	443 [418–497] (*n* = 34)	0.62
Hb—mg/dL, mean ± SD	14.2 ± 1.5 (*n* = 108)	14.1 ± 1.8 (*n* = 33)	0.82
Proteinuria ≥ (+), *n* (%)	19 (17) (*n* = 115)	7 (20) (*n* = 35)	0.63
Medication			
Aspirin allocation, *n* (%)	20 (17)	5 (14)	0.80
Insulin, *n* (%)	70 (60)	20 (57)	0.78
Sulfonylureas, *n* (%)	28 (24)	14 (40)	0.06
α-glucosidase inhibitors, *n* (%)	17 (15)	6 (17)	0.71
Biguanides, *n* (%)	8 (6.8)	1 (2.9)	0.69
Thiazolidines, *n* (%)	52 (44)	10 (29)	0.09
Calcium-channel blockers, *n* (%)	10 (8.6)	2 (5.7)	0.59
β-blockers, *n* (%)	3 (2.6)	3 (8.6)	0.14
α-blockers, *n* (%)	23 (20)	5 (14)	0.47
ACE inhibitors, *n* (%)	26 (22)	7 (20)	0.78
ARB, *n* (%)	1 (0.9)	0 (0)	1.00
Nitrate, *n* (%)	69 (59)	17 (49)	0.28
Antihypertensive drugs ≥ 1, *n* (%)	39 (33)	9 (26)	0.40
Antihypertensive drugs ≥ 2, *n* (%)	23 (20)	12 (34)	0.07
Statin, *n* (%)	14 (12)	0 (0)	0.32

ACE, angiotensin converting enzyme; ARB, angiotensin receptor blocker; BP, blood pressure; BMI, body mass index; BUN, blood urea nitrogen; CI, cerebral infarction; CRP, C-reactive protein; DM, diabetes mellitus; eGFR, estimated glomerular filtration rate; Hb, hemoglobin; HDL, high-density lipoprotein; ICH, intracerebral hemorrhage; IQR, interquartile range; LDL, low-density lipoprotein; RBC, red blood cell; SD, standard deviation; WBC, white blood cell.

**Table 4:** BP exposure measures.

BP Exposure Measures	CI Case (*n* = 117)	CI Control (*n* = 234)	*p* Value
The interval between recent BP measurement to onset-days, median [IQR]	300 [144–509]	288 [120–514]	0.46
Systolic BP before onset—mmHg, mean ± SD	135 ± 17	133 ± 14	0.15
Mean difference in systolic BP before onset between cases and matched controls—mmHg, (95% confidence interval)	2.4 (−1.3–6.1)		
Diastolic BP before onset—mmHg, mean ± SD	74 ± 11	74 ± 10	0.85
Mean difference in diastolic BP before onset between cases and matched controls—mmHg, (95% confidence interval)	−0.1 (−2.3–2.2)		
Mean BP before onset—mmHg, mean ± SD	95 ± 11	94 ± 10	0.51
Mean difference in mean BP before onset between cases and matched controls—mmHg, (95% confidence interval)	0.8 (−1.7–3.2)		
Median number of BP measurements during follow-up, median [IQR]	5 [2–7]	5 [2–7]	0.89
Coefficient of variation for systolic BP during follow-up, mean ± SD	0.07 ± 0.04 (*n* = 94)	0.07 ± 0.04 (*n* = 188)	0.88
Mean difference in coefficient of variation for systolic BP during follow-up between cases and matched controls (95% confidence interval)	0.001 (−0.01–0.01)		
Coefficient of variation for diastolic BP during follow-up, mean ± SD	0.09 ± 0.05 (*n* = 94)	0.09 ± 0.05 (*n* = 118)	0.61
Mean difference in coefficient of variation for diastolic BP during follow-up between cases and matched controls (95% confidence interval)	−0.003 (−0.02–0.009)		
	**ICH Case** **(*n* = 35)**	**ICH Control** **(*n* = 70)**	
The interval between recent BP measurement to onset—days, median [IQR]	481 [317–612]	448 [322–584]	0.76
Systolic BP before onset—mmHg, mean ± SD	137 ± 18	134 ± 15	0.40
Mean difference in systolic BP before onset between cases and matched controls (95% confidence interval)	2.8 (−4.3–9.9)		
Diastolic BP before onset—mmHg, mean ± SD	75 ± 11	72 ± 10	0.17
Mean difference in diastolic BP before onset between cases and matched controls (95% confidence interval)	3.0 (−1.1–7.1)		
Mean BP before onset—mmHg, mean ± SD	96 ± 11	93 ± 10	0.18
Mean difference in mean BP before onset between cases and matched controls—mmHg, (95% confidence interval)	2.9 (−1.4–7.3)		
Median number of BP measurements during follow-up, median [IQR]	5 [2–8]	5 [2–7]	0.79
Coefficient of variation for systolic BP during follow-up, mean ± SD	0.08 ± 0.04 (*n* = 27)	0.08 ± 0.04 (*n* = 54)	0.86
Mean difference in coefficient of variation for systolic BP during follow-up between cases and matched controls (95% confidence interval)	0.002 (−0.02–0.02)		
Coefficient of variation for diastolic BP during follow-up, mean ± SD	0.09 ± 0.04 (*n* = 27)	0.10 ± 0.05 (*n* = 54)	0.56
Mean difference in coefficient of variation for diastolic BP during follow-up between cases and matched controls (95% confidence interval)	−0.007 (−0.03–0.02)		

BP, blood pressure; CI, cerebral infarction; ICH, intracerebral hemorrhage; IQR, interquartile range; SD, standard deviation.

## Data Availability

The data that support the findings of this study are available from the corresponding author on reasonable request.

## Supplementary Materials

The following supporting information can be downloaded at: https://www.mdpi.com/article/10.3390/jcdd13100493/s1, Table S1: Sensitivity analyses of BP exposure measures excluding patients with two BP measurements.

## Abbreviations

The following abbreviations are used in this manuscript:

BP	Blood Pressure
CI	Cerebral Infarction
CV	Coefficient of Variation
ICH	Intracerebral Hemorrhage
JPAD	Japanese Primary Prevention of Atherosclerosis with Aspirin for Diabetes
