# Supplementary material for: Association Between Long-Term Blood Pressure Patterns and Onset of Stroke: A Nested Case–Control Study of a Randomized Clinical Trial
Source: J Cardiovasc Dev Dis. 2026 Oct 1;13(10):493. doi: 10.3390/jcdd13100493 (PMC13705313; doi:10.3390/jcdd13100493)
Supplement: Supplementary file 1 [file jcdd-13-00493-s001.zip › jcdd-4465073-supplementary.pdf]

Supplementary Material

Association between long-term blood pressure patterns and onset of stroke: A nested case-control study of a randomized clinical trial

Masataka Miyama, Fumihiro Sakakibara, Hisao Ogawa, Hirofumi Soejima, Sadanori Okada, Izuru Masuda, Masako Waki, Hideaki Jinnouchi, Yoshihiko Saito, Takeshi Morimoto

Table of contents

2 Supplementary Table S1. Sensitivity analyses of BP exposure measures excluding patients with two BP measurements

Supplementary Table S1. Sensitivity analyses of BP exposure measures excluding patients with two BP measurements

BP exposure measures	CI Case (*n* = 81)	CI Control (*n* = 162)	*p* Value
The interval between recent BP measurement to onset -days, median [IQR]	312 [154–555]	306 [168–560]	0.80
Systolic BP before onset -mmHg, mean ±SD	133 ±17	132 ±14	0.42
Mean difference of systolic BP before onset between cases and matched controls (95% confidence interval)	1.6 (-3.2 – 6.4)		
Diastolic BP before onset -mmHg, mean ±SD	73 ±11	73 ±9.4	0.83
Mean difference of diastolic BP before onset between cases and matched controls (95% confidence interval)	-0.3 (-3.1 – 2.5)		
Mean BP before onset -mmHg, mean ±SD	93 ±11	92 ±9.5	0.80
Mean difference in mean BP before onset between cases and matched controls -mmHg, (95% confidence interval)	0.3 (-2.7 – 3.4)		
Median number of BP measurements during follow-up, median [IQR]	6 [5–8]	6 [5–8]	0.84
Coefficient variation of systolic BP during follow-up, mean ±SD	0.08 ±0.004	0.07 ±0.003	0.68
Mean difference in coefficient of variation for systolic BP during follow-up between cases and matched controls (95% confidence interval)	0.002 (-0.009 – 0.01)		
Coefficient variation of diastolic BP during follow-up, mean ±SD	0.09 ±0.05	0.09 ±0.04	0.93
Mean difference in coefficient of variation for diastolic BP during follow-up between cases and matched controls (95% confidence interval)	-0.0005 (-0.01 – 0.01)		

BP exposure measures	ICH Case (*n* = 26)	ICH Control (*n* = 52)	*p* Value
The interval between recent BP measurement to onset -days, median [IQR]	464 [327–581]	464 [328–580]	1.00
Systolic BP before onset -mmHg, mean ±SD	137 ±14	133 ±14	0.32
Mean difference of systolic BP before onset between cases and matched controls (95% confidence interval)	3.4 (-3.6 – 10.4)		
Diastolic BP before onset -mmHg, mean ±SD	77 ±11	72 ±11	0.055
Mean difference of diastolic BP before onset between cases and matched controls (95% confidence interval)	5.0 (0.7 – 9.3)		
Mean BP before onset -mmHg, mean ±SD	97 ±10	92 ±10	0.07
Mean difference in mean BP before onset between cases and matched controls -mmHg, (95% confidence interval)	4.5 (-0.04 – 9.0)		
Median number of BP measurements during follow-up, median [IQR]	7 [5–9]	6 [4–8]	0.66
Coefficient variation of systolic BP during follow-up, mean ±SD	0.08 ±0.04	0.08 ±0.04	0.93
Mean difference in coefficient of variation for systolic BP during follow-up between cases and matched controls (95% confidence interval)	0.0008 (-0.02 – 0.02)		
Coefficient variation of diastolic BP during follow-up, mean ±SD	0.09 ±0.04	0.09 ±0.05	0.78
Mean difference in coefficient of variation for diastolic BP during follow-up between cases and matched controls (95% confidence interval)	-0.003 (-0.03 – 0.02)		

BP, blood pressure; CI, cerebral infarction; ICH, intracerebral hemorrhage; IQR, interquartile range; SD, standard deviation.
